# The Role of H3K4me3 in Transcriptional Regulation Is Altered in Huntington’s Disease

**DOI:** 10.1371/journal.pone.0144398

**Published:** 2015-12-04

**Authors:** Xianjun Dong, Junko Tsuji, Adam Labadorf, Panos Roussos, Jiang-Fan Chen, Richard H. Myers, Schahram Akbarian, Zhiping Weng

**Affiliations:** 1 Program in Bioinformatics and Integrative Biology, University of Massachusetts Medical School, Worcester, MA, United States of America; 2 Department of Neurology, Boston University School of Medicine, Boston, MA, United States of America; 3 Bioinformatics Program, Boston University, Boston, MA, United States of America; 4 Friedman Brain Institute, Department of Psychiatry, Mount Sinai School of Medicine, New York, NY, United States of America; 5 Department of Genetics and Genomic Sciences, Mount Sinai School of Medicine, New York, NY, United States of America; 6 Genome Science Institute, Boston University School of Medicine, Boston, MA, United States of America; Ludwig-Maximilians-Universität München, GERMANY

## Abstract

Huntington’s disease (HD) is an autosomal-dominant neurodegenerative disorder resulting from expansion of CAG repeats in the Huntingtin (*HTT*) gene. Previous studies have shown mutant *HTT* can alter expression of genes associated with dysregulated epigenetic modifications. One of the most widely studied chromatin modifications is trimethylated lysine 4 of histone 3 (H3K4me3). Here, we conducted the first comprehensive study of H3K4me3 ChIP-sequencing in neuronal chromatin from the prefrontal cortex of six HD cases and six non-neurologic controls, and its association with gene expression measured by RNA-sequencing. We detected 2,830 differentially enriched H3K4me3 peaks between HD and controls, with 55% of them down-regulated in HD. Although H3K4me3 signals are expected to be associated with mRNA levels, we found an unexpected discordance between altered H3K4me3 peaks and mRNA levels. Gene ontology (GO) term enrichment analysis of the genes with differential H3K4me3 peaks, revealed statistically significantly enriched GO terms only in the genes with down-regulated signals in HD. The most frequently implicated biological process terms are organ morphogenesis and positive regulation of gene expression. More than 9,000 H3K4me3 peaks were located not near any recognized transcription start sites and approximately 36% of these “distal” peaks co-localized to known enhancer sites. Six transcription factors and chromatin remodelers are differentially enriched in HD H3K4me3 distal peaks, including EZH2 and SUZ12, two core subunits of the polycomb repressive complex 2 (PRC2). Moreover, PRC2 repressive state was significantly depleted in HD-enriched peaks, suggesting the epigenetic role of PRC2 inhibition associated with up-regulated H3K4me3 in Huntington’s disease. In summary, our study provides new insights into transcriptional dysregulation of Huntington’s disease by analyzing the differentiation of H3K4me3 enrichment.

## Introduction

Huntington’s disease (HD) is an autosomal-dominant neurodegenerative disorder characterized by abnormal involuntary choreiform movements, cognitive impairment, and psychiatric dysfunction [1,2]. Neuropathologically, HD patients exhibit neuronal cell loss and gliosis, primarily in the striatum, but also involving the cerebral cortex and other brain regions [3,4]. Although there are currently no effective treatments for persons afflicted with HD, recent trials of creatine monohydrate among asymptomatic HD gene carriers suggest it may have potential to delay the disease onset [5]. The cause of HD is an expansion in the number of CAG trinucleotide repeats in the coding region of exon 1 of the Huntingtin (*HTT*) gene [6]. In comparison to the normal *HTT* allele, which ranges from 8 to 35 triplet repeats, mutant *HTT* (*mHTT*) contains 36 or more repeats resulting in an expanded polyglutamine tract [6]. Repeats between 36 and 39 units show reduced penetrance [7,8].

The toxic *mHTT* expansion induces numerous and widespread aberrant molecular effects. Transcriptional dysregulation has been proposed to be a central component of HD pathogenesis [9,10]. Altered gene expression has been reported [11] and several studies support alterations at one or more of the stages of RNA processing, translation, protein post-translational modification and trafficking [12–14]. Previous studies show that mHTT protein physically binds CREB-binding protein (CBP) and blocks both CBP’s transcription coactivator function in human and mice [15] and its histone acetyltransferase activity in Drosophila [16]. Studies in mouse models of HD further illustrate that transcriptional dysfunction is associated with histone hypoacetylation [17,18]. Elevated expression of ERG-associated protein with SET domain (ESET) in HD patients and in the R6/2 transgenic mouse model increases abnormal trimethylation of histone H3 lysine 9 (H3K9me3) [19]. The decreased expression of acetylcholine receptor 1 (CHRM1) in HD also affects H3K9me3-mediated chromatin remodeling [20]. Interestingly, the gene expression changes in HD are usually associated with dysregulated epigenetic modifications [21–23].

One of the most widely studied histone modifications is trimethylated lysine 4 of histone 3 (H3K4me3) [24,25]. H3K4me3 marks active transcription start sites (TSSs) [26–28] and the strength of H3K4me3 signal at the promoter is strongly correlated with the expression of the gene [29,30]. Recently, studies in the R6/2 HD transgenic mouse model revealed that H3K4me3 may play a critical role in the pathway leading to transcriptional dysregulation in HD [31]. Our earlier study found that epigenetic changes in the *HES4* gene are associated with reduced H3K4me3 and increased DNA methylation in its promoter, and showed that these changes were closely correlated with degeneration in the striatum of HD patients [32]. These previous findings motivated us to investigate the genome-wide pattern of H3K4me3 in HD. In the current study, we conducted the first chromatin immunoprecipitation using anti-H3K4me3 antibody followed by sequencing (ChIP-seq) experiments on NeuN-selected neuronal cell nuclei from post-mortem prefrontal cortical samples for six HD cases and six non-neurologic controls.

## Results

### H3K4me3 modification enrichment characteristics

We analyzed H3K4me3 enrichment in FACS sorted neuronal nuclei derived from postmortem prefrontal cortex (BA9) of six HD cases and six non-neurological controls (Table 1). A total of 28,608 H3K4me3 peaks were defined when the filtered peaks called from HD and control samples were combined (S1 Fig). We found that most peaks (59.2%) were called in all twelve samples (S2A Fig) and on average 63% of total H3K4me3 ChIP-seq reads were mapped to TSS-proximal peaks. These are consistent with our prior data showing that H3K4me3 is enriched in promoters and similar across different individuals [33–35]. The H3K4me3 signal tends to be stronger when the peak is called in more samples (S2B Fig).

**Table 1:** Characteristics of human brain samples in the study.

Diagnosis	ID*	Sex	CAG Repeat	Onset age	Age at death	PMI^+^	Antibody	Total reads	Uniquely mapped reads	Mappability (%)	Peaks
HD	H0001	M	45	44	55	37.25	H3K4me3	6,134,681	5,206,490	84.90%	27,661
HD	H0003	M	43	52	71	20.5	H3K4me3	3,292,319	2,930,762	89.00%	24,955
HD	H0008	M	49	28	43	21.3	H3K4me3	6,647,419	5,979,404	90.00%	29,710
HD	H0009	M	42	45	68	3.73	H3K4me3	2,817,246	2,540,805	90.20%	29,640
HD	H0681	M	42	50	69	19.06	H3K4me3	5,567,462	4,858,701	87.30%	27,732
HD	H0601	F	45	unknown	56	19.0	H3K4me3	5,943,156	5,282,199	88.90%	30,647
Control	C1	M	N/A	N/A	55	17	H3K4me3	6,028,542	4,927,721	81.70%	40,150
Control	C2	M	N/A	N/A	63	24.5	H3K4me3	8,356,841	7,519,896	90.00%	30,434
Control	C3	M	N/A	N/A	64	28.21	H3K4me3	13,238,359	11,132,414	84.10%	27,419
Control	C4	M	N/A	N/A	74	12	H3K4me3	3,091,666	2,581,032	83.50%	39,323
Control	C5	M	N/A	N/A	81	8	H3K4me3	10,313,831	7,955,599	77.10%	32,907
Control	C6	F	N/A	N/A	68	7	H3K4me3	8,287,623	5,993,036	72.30%	38,970
Control	C7	F	N/A	N/A	69	40.15	Input	15,875,168	12,246,790	77.10%	1,616

*IDs match those for the HD samples that we published previously [12,36] (Labadorf et al. submitted);

^+^PMI: postmortem interval.

**Fig 1:**
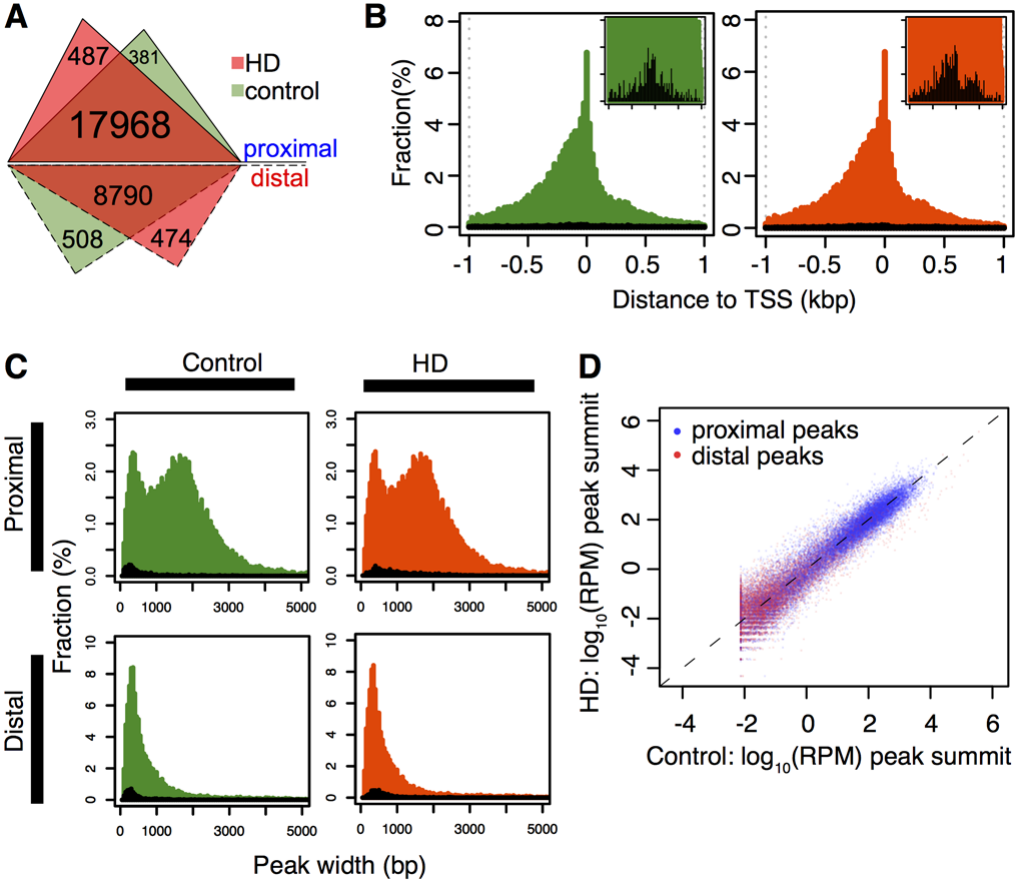
H3K4me3 proximal and distal peaks in HD and control. **(A)** The number of H3K4me3 peaks in HD and control. 487 proximal peaks were unique to HD samples while 381 were unique to controls. 474 distal peaks were unique to HD samples and 508 were unique to controls. (**B**) Histograms of the position of proximal peaks relative to the TSS in HD and control show a slight enrichment upstream the TSS. Small right panels show zoomed-in histograms for unique proximal peaks (highlighted in black). (**C**) Histograms of the distribution of peak widths for proximal and distal peaks were similar in control and HD samples. Proximal peaks show a bimodal distribution in peak width not seen in distal peaks. The width distributions of unique peaks are in black. (**D**) Peak intensities of proximal (blue) and distal (red) peaks are plotted for HD versus control.

Most H3K4me3 peaks (n = 18,836, 65.8%) were located proximally (within 1 kb) to a TSS and most (93.5%) were shared between HD and control, with 6.5% peaks (n = 1,850) unique to HD or control (Fig 1A). Proximal peaks were positioned with a slight upstream position bias relative to TSSs, in both control and HD samples (Fig 1B), which was seen for both common and uniquely defined proximal peaks. The 9,772 distal peaks (>1 kb from TSS) consist of 5,671 (58.0%) are from intragenic regions of annotated genes and 4,102 (42.0%) from intergenic regions. Both proximal and distal peaks were confidently mapped with strong read depth (Fig 1D).

Among proximal peaks, the distribution of peak widths was bimodal with the two modes being 350 bp and 1700 bp (Fig 1C). The proximal peaks unique to HD are slightly larger (t-test p-value = 3.28x10^-14^) than those unique to controls (400bp versus 300bp respectively). The mode for distal peaks is 350bp, similar to the mode for the smaller proximal peaks.

### Differential H3K4me3 between HD and control

To investigate the transcript specific differences in H3K4me3 signal, we extracted H3K4me3 reads within a 2000 bp window centered around every annotated TSS (referred to as promoters hereafter) regardless of whether a peak was called within that region or not. This strategy permits an assessment of the relationship of H3K4me3 signal to mRNA-seq abundance (see Method). As a result, we detected 720 genes with significantly different H3K4me3 level in promoters between HD and control, including 104 genes with significantly higher H3K4me3 signal in HD and 616 genes with significantly higher H3K4me3 in controls (S3 Table). We also ran the differential analysis for all 9,772 distal peaks, and detected 209 and 51 distal peaks with significantly higher H3K4me3 signal in HD and control respectively (S4 Table).

Table 2 lists the 22 genes that have the most differential H3K4me3 signal at the proximal promoter regions between HD and control (q-value< 1x10^-4^). Among them 15 genes have lower H3K4me3 in HD cases than in controls. *HES4*, which we previously reported [32], is the fourth protein-coding gene in this list. The top gene *ALG14* catalyzes asparagine (N)-glycosylation, which is an essential modification that regulates protein folding and stability.

**Table 2:** Genes that correspond to the top 22 differential proximal H3K4me3 peaks.

Gene symbol	Gene type	log_2_(HD/control)	q-value	Gene / Function
*ALG14*	protein coding	1.028	3.63E-09	UDP-GlcNAc transferase: regulates protein folding and stability
*SERTAD4-AS1*	Antisense	-1.075	1.02E-07	Antisense of SERTAD4: no known function
*LRRTM2*	protein coding	1.110	1.08E-06	leucine rich repeat transmembrane neuronal 2: development of excitatory synapse in CNS
*CTD-3064M3*.*4*	processed transcript	-1.091	1.96E-06	N/A
*FAM184B*	protein coding	-1.067	2.28E-06	No known function
*HES4*	protein coding	-1.506	1.30E-05	Hairy and enhancer of split 4: protein dimerization & transcription factor binding.
*CTB-31O20*.*8*	antisense	-1.347	1.43E-05	N/A
*TMEM61*	protein coding	-1.958	1.53E-05	Transmembrane Protein 61: no known function
*SNORD118*	snoRNA	1.464	2.19E-05	Small Nucleolar RNA, C/D Box 118, predicted to serve as guide RNAs in ribose methylation of rRNA.
*COX7B*	protein coding	1.294	2.24E-05	Cytochrome C oxidase subunit 7B: Mitochondrial, terminal component of respiratory chain
*RP11-783K16*.*10*	processed transcript	-1.007	2.43E-05	N/A
*MIR5188*	miRNA	-1.072	2.84E-05	N/A
*RAMP3*	protein coding	-1.198	3.78E-05	Receptor activity modifying protein 3: transport calcitonin-receptor-like-receptor to plasma membrane
*AC092171*.*1*	lincRNA	-1.075	3.88E-05	N/A
*RP11-982M15*.*5*	antisense	-1.738	3.88E-05	N/A
*RP11-44N21*.*4*	processed transcript	-1.067	3.95E-05	N/A
*RP5-1120P11*.*3*	lincRNA	-1.126	4.65E-05	N/A
*SNORD50B*	snoRNA	1.310	4.99E-05	Small nucleolar RNA, C/D box 50B, predicted to guide 2´-O-methylation of 28S rRNA
*DSG2*	protein coding	-1.592	6.23E-05	Desmoglein 2, cadherin family member 5: calcium ion binding
*SNORD13*	snoRNA	1.102	7.15E-05	Small nucleolar RNA, C/D Box 13: no predicted function
*PALD1*	protein coding	-1.190	8.66E-05	Phosphatase domain containing paladin 1: no known function
*FLRT3*	protein coding	1.033	9.09E-05	Fibronectin leucine rich transmembrane protein 3: receptor signaling protein activity and protein binding, bridging

Note: N/A means either the protein’s function is uncharacterized or there is no literature found on the function of the gene.

### Proximal H3K4me3 signal is correlated with gene expression

**Fig 2:**
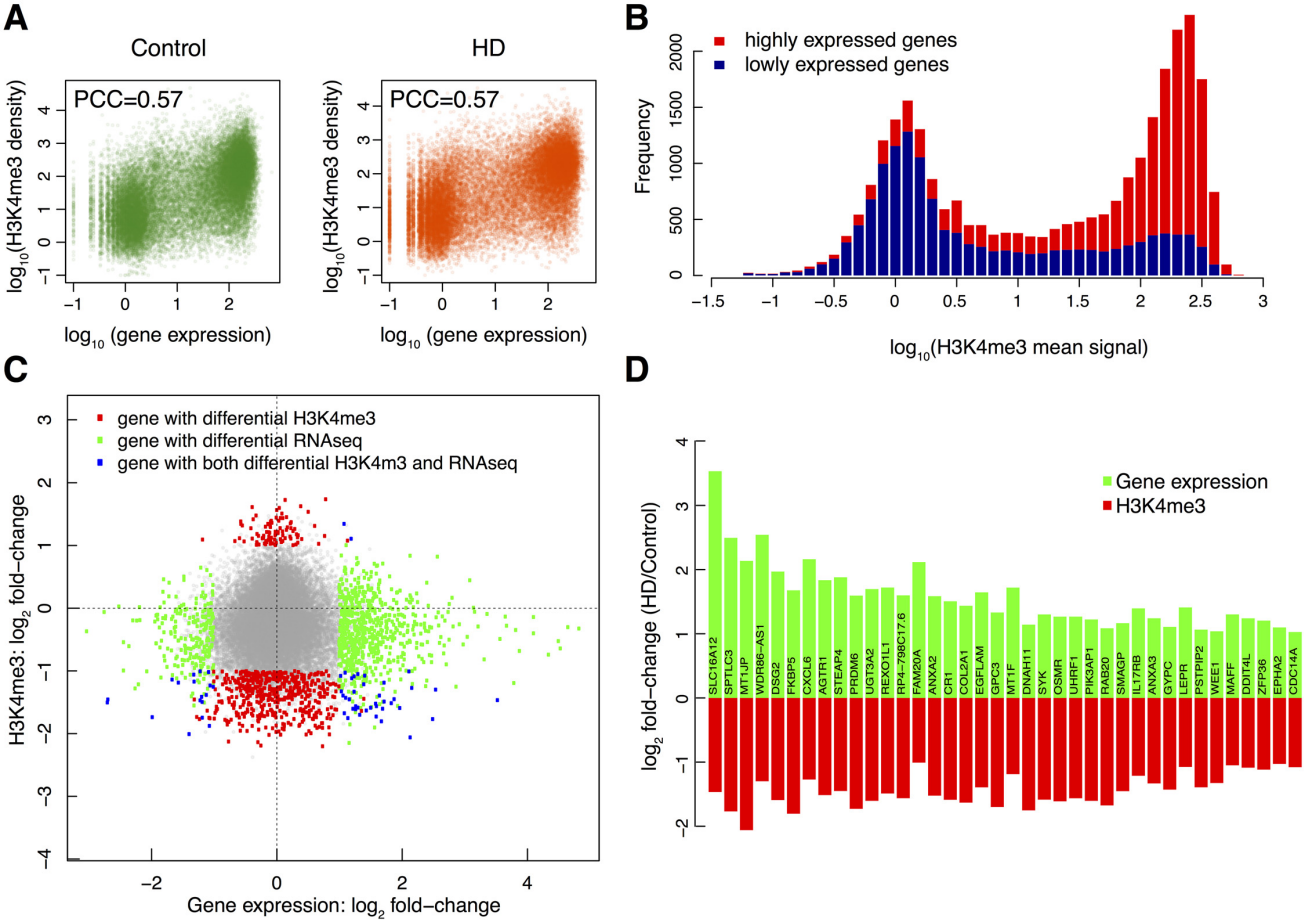
Gene expression and H3K4me3 signal at promoters. **(A**) Correlation of H3K4me3 signal at promoters and gene expression in control and HD. X-axis shows the logarithmic base mean value of normalized expression values CPKM (RNA-seq reads count per kilobase of exon length per millions of total mapped reads), and Y-axis shows the logarithmic signal density of ChIP-seq reads at proximal promoters (i.e. [-1k, 1k] of TSS). It shows that gene expression levels are positively correlated with the density of H3K4me3 signal at promoters in both controls and HD, with Spearman correlation rho = 0.57. (**B**) Histogram of normalized H3K4me3 signal in all samples. Bars are colored according to the frequency of genes with high (red) and low (blue) expression. (**C**) Scatter plot of log_2_(fold change between HD and control) of H3K4me3 signal at promoter versus gene expression. Genes with differential expression but not differential H3K4me3 are in green; genes with differential H3K4me3 but not differential expression are in red, and genes with both differential expression and H3K4me3 are in blue. In total, 58 genes show both differential expression and H3K4me3, where only 20 of them show the same direction of differentiation between control and HD cases. The remainder (38 of 58) shows a lack of concordance between differential expression and differential H3K4me3 at promoter. **(D)** Fold changes of expression (in green) and H3K4me3 (in red) between HD and control for the 38 genes with lack of concordance.

We calculated the correlation between the signal at proximal H3K4me3 peaks and the expression level of the corresponding genes. Even though we performed both H3K4me3 ChIP-seq and RNA-seq experiments on brain tissue from the BA9 region of the prefrontal cortex, ChIP-seq was performed on sorted neuronal nuclei while RNA-seq was performed on brain tissue homogenate because it is not feasible to sort entire cells. Previously we showed that H3K4me3 levels at promoters were highly correlated with transcription levels of genes across multiple cell types [37]. We confirmed this pattern in both control and HD cases (Spearman’s correlation coefficient *rho* = 0.57, p-value < 2.2e-16, see Fig 2A). In addition, because of the bimodality seen for both the mRNA expression (S3A Fig) and H3K4me3 level (S3B Fig) for both HD and control, we subdivided genes into high and low groups by gene expression and H3K4me3 level respectively. Applying a χ² test, we found that genes with high and low H3K4me3 levels strongly associate with high and low expression respectively (χ^2^ p-value < 2.2 × 10^−16^; see Fig 2B for all samples). This relationship was seen in both controls and HD cases (S3C Fig).

We evaluated whether differentially expressed genes (DEGs) between HD and control also exhibit differential H3K4me3 levels at their promoters. Most genes are neither differentially expressed nor have differential H3K4me3 signal at the promoters. Among the 961 DEGs and the 720 genes with differential H3K4me3 signals, 58 genes were found to have both differential expression and H3K4me3 (blue dots in Fig 2C). No correlation was found between the fold change of differential expression and that of differential H3K4me3 (Spearman *rho* = 0.04, Fig 2C). Out of the 58 genes, 20 showed the same direction of alteration; 18 cases of both decreased expression and H3K4me3 levels in HD and only two cases of both increased genes and H3K4me3 in HD. The trend that the majority of the dysregulated genes are down-regulated is consistent with a previous study in the HD mouse R6/2 model [31]. It is worth noting that the remaining 38 genes are in the opposite direction; increased gene expression but decreased H3K4me3 in HD (as listed in Fig 2D). These include calcium-signaling genes (e.g. *DSG2*, *ANXA2*, and *ANXA3*), as well as protein kinase or kinase receptors (e.g. *WEE1*, *OSMR*, and *IL17RB*). Interestingly, epigenetic regulators are also found, such as *PRDM6* (histone methyltransferase) and *UHRF1* (which can recruit the DNA methyltransferase *DNMT1* to regulate chromatin structure and gene expression). Several genes involved in neuronal function are seen. For instance, *EGFLAM* (gene for the Pikachurin protein) is known to play important role in the retinal photoreceptor ribbon synapse formation [38], and *EPHA2*, a kinase receptor, plays multiple roles in neural development [39,40].

### GO terms enriched in the genes with proximal H3K4me3 peaks

**Fig 3:**
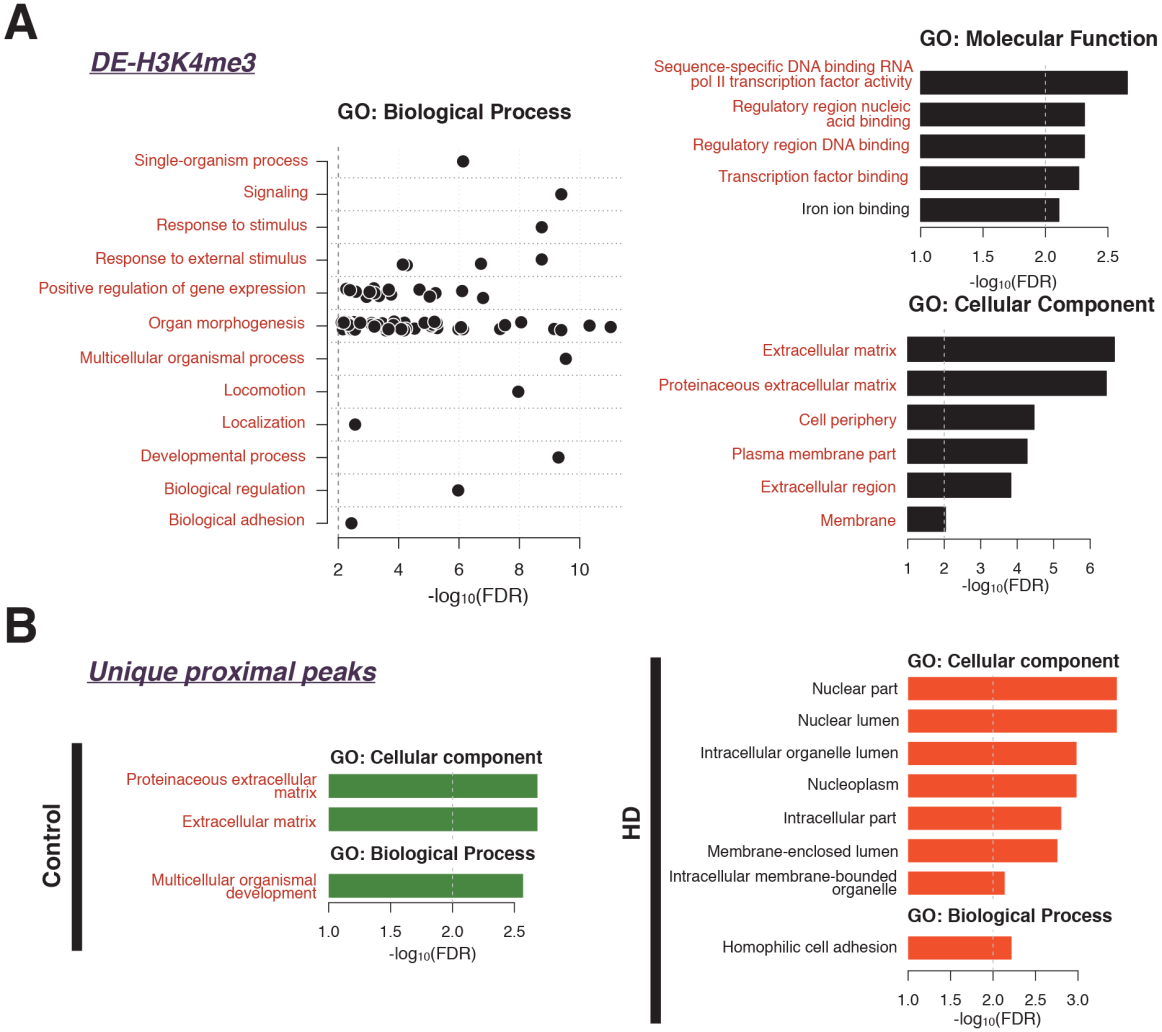
GO terms enriched with proximal peaks in control and HD. **(A)** GO terms enriched with genes marked with down-regulated H3K4me3 signals in HD. The GO terms overlapped with those derived from differentially expressed genes are highlighted in red text, with iron ion binding (in black) representing the only term not seen in the differentially expressed genes. X-axis shows the negative logarithm FDR values. **(B)** GO terms enriched with genes marked with unique proximal peaks in HD (right; orange) and in control (left; green). These terms also overlap with those seen in HD differentially down-regulated genes.

We computed the enriched GO terms of the genes with 720 proximal differential H3K4me3 signals; 104 up-regulated and 616 down-regulated in HD. We only detected GO terms significantly enriched in the 616 genes with lower H3K4me3 signals in HD than in control. The non-redundant GO terms are shown in Fig 3A. Although the biological process GO terms were diverse, organ morphogenesis and positive regulation of gene expression were the most frequently implicated terms. The molecular function and cellular component GO terms were related to transcription and the extracellular matrix.

We also calculated enriched GO terms of the 961 DEGs; 718 up-regulated and 243 down-regulated in HD. Contrary to the differential H3K4me3 results, all detected 664 GO terms were those enriched with up-regulated genes; 576 terms in biological processes, 51 terms in molecular functions and 37 terms in cellular components (S4 Fig).

Interestingly, the GO terms enriched with the genes marked with down-regulated H3K4me3 signals resembled or overlapped with the GO terms enriched with up-regulated genes as parent-child relationships in the GO hierarchical structure (Fig 3A, S4 Fig). Except for iron ion binding (GO:0005506) in molecular function terms, all of the GO terms enriched with the genes with down-regulated H3K4me3 signals were covered by those enriched with up-regulated genes.

GO terms enriched with the genes with unique proximal peaks are presented; 381 and 487 peaks in control and HD respectively. As seen in Fig 3B, all GO terms enriched with genes marked with unique proximal peaks in control overlapped with those for genes marked with down-regulated peaks in HD (multicellular organismal development (GO:0007275) in Fig 3B is included in multicellular organismal process (GO:0032501) in Fig 3A). In HD, interestingly GO terms related to lumen (nuclear lumen (GO:0031981), intracellular organelle lumen (GO:0070013), and membrane-enclosed lumen (GO:0031974)), were detected. The genes for which unique HD proximal peaks are found at their TSS, tend to represent nuclear, organelle, and intracellular components. Among the GO terms for biological processes, homophilic cell adhesion (GO:0007156) was detected for genes with unique H3K4me3 proximal peaks.

### Networks of the genes with differential proximal H3K4me3 peaks

We conducted pathway analysis with the SPIA tool [41] and mapped the genes with differential H3K4me3 on biological pathways. We detected eleven significant pathways (see S5 Table), and five of these are signaling pathways (Rap1, PI3K-Akt, cAMP, Hippo, and Calcium). Consistent with our GO analysis above indicating that many genes with down-regulated H3K4me3 in HD are enriched in extracellular or membrane GO terms, our pathway analysis found that these genes tend to be clustered at the cellular membrane and located in the upper ranks in the signaling pathways and the extracellular matrix (ECM)-receptor interaction pathway.

We also calculated the enriched pathways for DEGs. We detected 25 significant pathways and found that most genes in these pathways were up-regulated in HD (see S6 Table). The PI3K-Akt and Hippo signaling pathways were also detected to be enriched. As reported by Labadorf et al. (submitted), many of the pathways were related to immune function.

**Fig 4:**
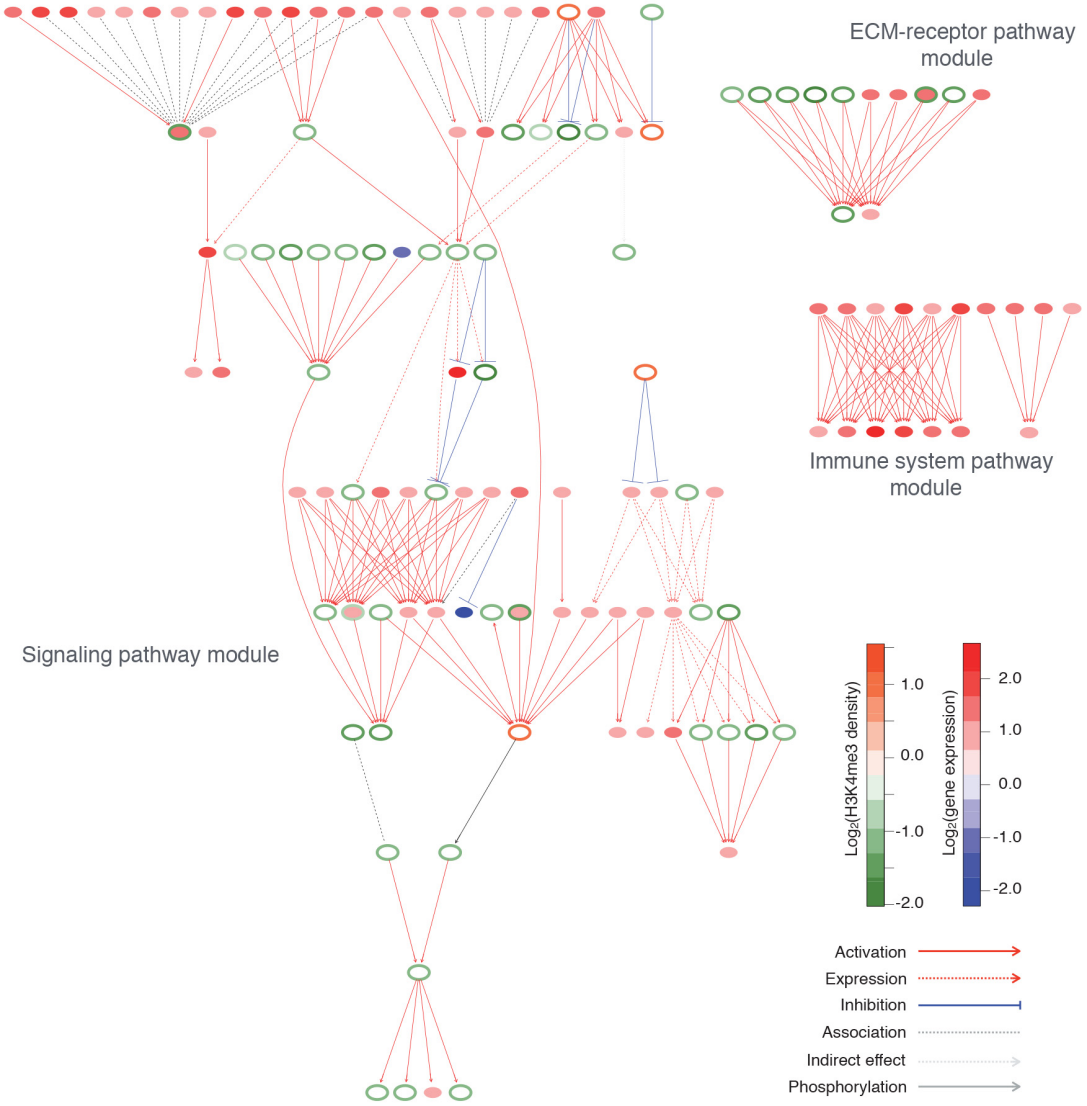
Sub-networks of DEGs and genes with differential H3K4me3. Each node represents a gene; the fill color of each node indicates the fold-change of gene expression between HD and control, and the outline color of each node indicates the fold-change of H3K4me3 between HD and control. Immune system and ECM-receptor pathway modules were composed of specific gene families. On the other hand, signaling pathway modules were composed of various signaling genes forming hierarchical structure. The directions of all the arrows were drawn toward the bottom of figure.

To investigate the relationships between the genes with differential H3K4me3 and DEGs, we extracted the gene interaction sub-networks of the detected pathways in S5 and S6 Tables. The gene interaction annotation used in this analysis is also based on the annotation in KEGG database. All genes in significant pathways are summarized in S7 Table. As we observed in the above pathway gene enrichment analysis, the two gene sub-networks of DEGs were extracted as the immune system module (Fig 4). Each sub-network was composed of a specific gene family. One of the two immune system sub-networks was formed by chemokine receptors (CCR1, 5, and 9; CXCR1, 2, and 4) and ligands (CCL2, and 26; CXCL1, 5, 6, and 7), and the other was formed by complement subcomponents (C1QA, C1QB, C1QC, C1R, and C4B).

On the other hand, the sub-network of the genes with differential H3K4me3 was mainly detected as the signaling pathway module (Fig 4) and was composed of various signaling genes listed in S7 Table. Those genes in the signaling pathway module straddled across the cell membrane to the inside of the cell and preserved cascade structures in the pathways detected in the above pathway gene enrichment analysis. Although several DEGs were also included in the signaling pathway module, those DEGs were in a specific gene family (e.g. immunoglobulin-like receptor gene family in the upstream of the signaling pathway module) and were not the hubs in the module. That is, even if we remove the DEGs from the signaling pathway module, still the hierarchical structure composed of the genes with differential H3K4me3 remains in the module. We also found the ECM-receptor pathway module composed of 12 extracellular component genes such as collagen and laminin.

### Evidence for regulatory role of distal H3K4me3 peaks

**Fig 5:**
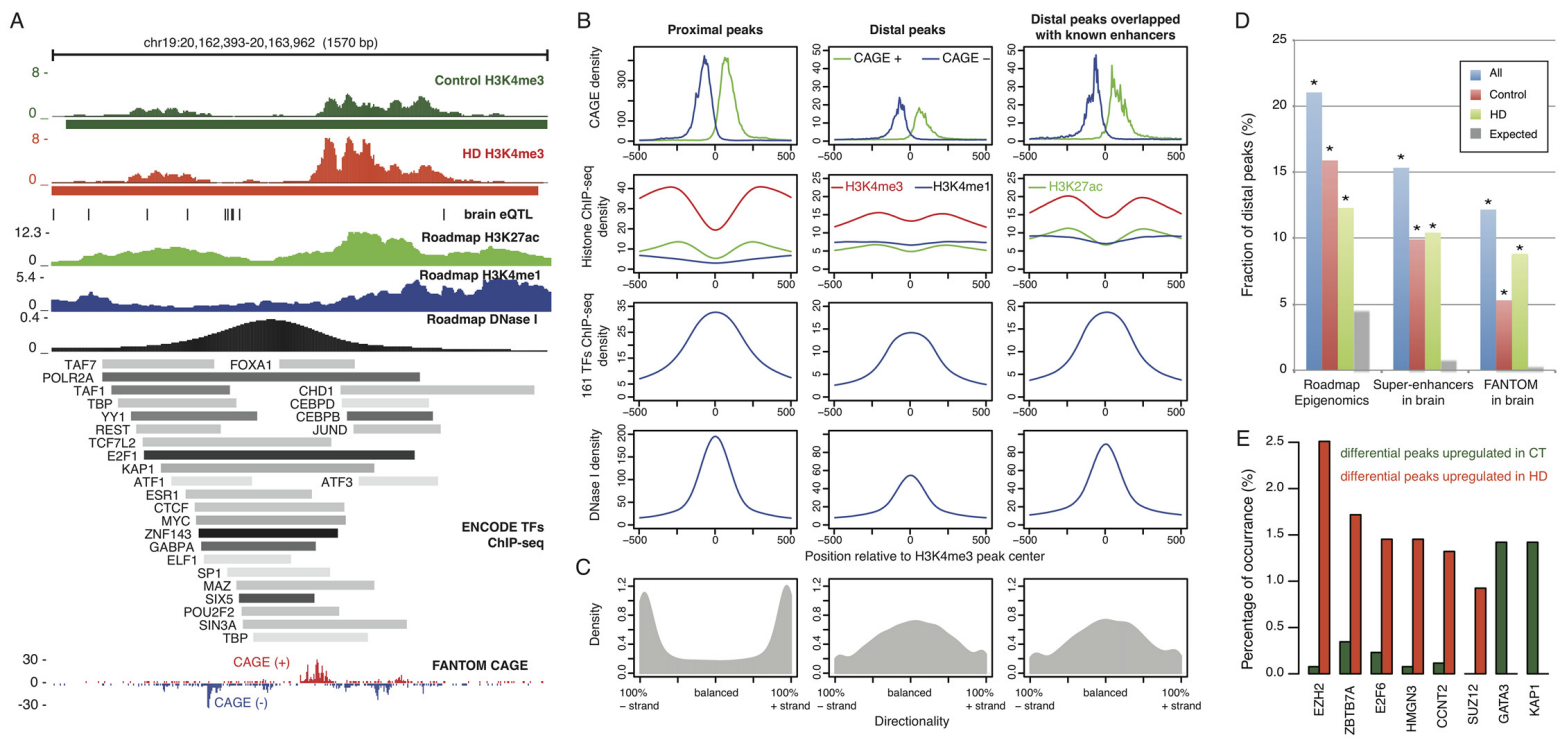
Overlapped regulatory signals and differentially enriched TF binding on distal peaks. **(A)** UCSC genome browser of a genomic locus (coordinate in hg19 shown on the top) where a distal H3K4me3 peak, called in both control and HD cases, overlaps with enhancer regulatory features including enriched H3K4me1 (“Roadmap H3K4me1”), H3K27ac (“Roadmap H3K27ac”), DNase I (“Roadmap DNase I”), TFs binding hotspot (“ENCODE TFs ChIP-seq”), and bidirectional CAGE signals (“FANTOM CAGE”). **(B)** Aggregation plots of several types of regulatory signals, including CAGE (1^st^ row), histone marks (2^nd^ row), TF binding (3^rd^ row), and DNase I hypersensitivity (4^th^ row), centered on the middle of H3K4me3 peaks. **(C)** Directionality of H3K4me3 peaks represented by CAGE tags shows that, unlike the strong bias towards sense strand for proximal peaks, distal peaks show similar level of CAGE tags in both directions. **(D)** Fraction of distal peaks overlapping with different sources of known enhancers occurred at rates greater than expected (All = blue, control = red, HD = green, Expected = grey). **(E)** Transcription factors binding in differentially enriched distal peaks.

One third of H3K4me3 peaks were localized at more than 1 kb away from any known TSS. To investigate their potential regulatory functions, we first assessed whether they overlapped with genomic regions with other regulatory signals. Recent progress in functional genomics has amassed many biochemical data for regulatory elements, including the co-occurring histone marks of H3K4me1 and K3K27ac [26], bidirectional CAGE signal at promoters [42], hotspot of transcription factor binding sites [43], clustered DNaseI hypersensitive sites [44], and enriched eQTLs [45]. By aligning these signals from prefrontal cortex and other cortical related brain regions with our H3K4me3 data, we found interestingly many of the distal H3K4me3 peaks co-localize with regulatory elements defined by these signals. For example, although the distal peak in Fig 5A was called in both HD and control samples, it is much stronger in HD. It harbors eleven SNPs that were shown to be eQTLs in brain (black vertical lines in Fig 5A). Notably, this region is also centered on ChIP-seq peak of enhancer marks of H3K27ac and H3K4me1 on human frontal cortex from the Roadmap Epigenomics project. Active enhancers often correspond to open chromatin regions, which facilitate binding of transcription factors. Indeed, this region has open chromatin (the DNase track in Fig 5A) and is bound by more than 20 transcription factors (ENCODE TFs ChIP-Seq track in Fig 5A) particularly those known key factors for facilitating the 3D structure of chromatin, such as CTCF [46] and SP1 [47,48]. Finally, CAGE, a technique for capturing the 5′ end of mature transcripts, was recently found useful for identifying active enhancers based on a bi-directional signal [42]. It showed clear bi-directional signals at this region and an enhancer was predicted by FANTOM5 consortium (CAGE track in Fig 5A). Furthermore, we also found that the role of distal peaks as enhancers is also supported by the aggregated signal of CAGE tags, H3K4me3, H3K9ac, and DNase I on all distal peaks (Fig 5B). Transcription directionality based on the CAGE data from the FANTOM5 project further supports this view (Fig 5C). Thus the distal differential H3K4me3 peaks may represent differential enrichment at enhancer sites.

By computing the percentage of distal peaks overlapping with three datasets of known enhancers (see Methods), we found that 15–22% of distal peaks significantly overlapped known enhancers (p-value < 2.2 × 10^−16^; Fig 5D). Altogether, an estimated 36% of distal peaks overlapped with at least one of three enhancer datasets (S4 Table). The aggregation signals of regulatory marks were even more enriched on this subset of distal peaks than the total set of distal peaks, as seen from the third column of Fig 5B. From the set of distal peaks that overlap with at least one of the three known enhancer sets, 74 enhancer-like distal peaks are differentially enriched between HD and control (S4 Table).

To investigate the potential functional roles of distal H3K4me3 peaks, we analyzed the TFBS enrichment at these peaks with the most recent TF ChIP-seq data from the ENCODE project (see Methods). We identified those TFs specifically binding to distal peaks that were differentially enriched between HD and control. By comparing the TF binding occurrences between 209 and 51 differential distal peaks that were up-regulated in HD and control respectively, we found that six transcription factors (ZBTB7A, E2F6, CCNT2, HMGN3, EZH2, and SUZ12) had enriched binding sites in HD-specific distal peaks and two TFs (GATA3 and KAP1) had enriched binding sites in control-specific distal peaks (Fig 5E). Among these, EZH2, a histone methyltransferase, had the most significant difference (adjusted p-value = 2.87x10^-10^).

Both EZH2 and SUZ12 are subunits of polycomb repressive complex 2 (PRC2), which can primarily methylate histone 3 on lysine 27 (H3K27me3) and therefore repress transcription. In this study, we further looked into the enrichment of Polycomb-repressed regions (indicated by the ‘ReprPC’ and ‘ReprPCWk’ ChromHMM states in human frontal cortex of the Roadmap Epigenomics project [49]) in HD-specific peaks. We found that the repressed Polycomb state, representing the H3K27me3 signal, is depleted in the distal peak regions with significantly enriched H3K4me3 signal in HD (hypergeometric test p-value = 4.66x10^-5^), but not in control-enriched distal peaks (hypergeometric test p-value = 0.39). Previous study had shown that H3K4me3 can inhibit the activity of PRC2 in an allosteric fashion assisted by the Su(z)12 C terminus [50]. Our result of PRC2 depletion in the regions with increased H3K4me3 in HD might suggest that the epigenetic role of PRC2 inhibition is caused by up-regulated H3K4me3 in Huntington’s disease.

## Discussion

While patterns of H3K4me3 have been previously studied in the R6/2 mouse model of HD [31], we believe this is the first genome wide study of this mark in human HD versus control brains. We had previously performed mRNA-sequencing in a series of 20 HD and 49 non-neurological control brains, and therefore, we were able to compare the patterns of H3K4me3 enrichment with 961 differentially expressed mRNAs (FDR q<0.01) in HD and control brains (Labadorf et al. submitted). Because 50% to 90% of neurons have degenerated in the HD striatum, even among low-grade HD cases [3], we chose to analyze the prefrontal cortex (Brodmann area 9). While the hallmarks of HD pathology (e.g. intranuclear inclusion bodies) are present in the prefrontal cortex, the extent of neuronal degeneration is considerably less severe than that seen in the striatum [3,51]. Some of the HD samples studied here have been previously studied by microRNA-sequencing and mRNA-sequencing of the same region of the prefrontal cortex [12,36] (Labadorf et al. submitted).

In this study, we sought to minimize the biases introduced by simple neurodegeneration in two ways. First, the H3K4me3 ChIP-sequencing was performed in NeuN+ sorted neuronal nuclei [35,52]. Thus, the contrast is specific to neurons in both HD and controls and accounts for possible differences in the densities of specific cell types in the HD brain due to degeneration or reactive gliosis. Second, the prefrontal cortex (BA9), a region with mild neuropathological involvement in HD [3,4], was selected for analysis. A total of 28,608 H3K4me3 peaks were defined in the combined HD and control samples. While most peaks (93.5%) were called in both HD and control brains and a majority (59.2%) were called in all twelve samples studied, 961 were uniquely called in HD brain and 889 were uniquely called in controls (Fig 1, S2A Fig). The H3K4me3 mark is known to mark active transcription start sites (TSS), and 65.8% of the called peaks were located within 1,000 bp of an annotated TSS (designated “proximal peaks”). Both the proximal peaks and distal peaks (positioned at greater than 1000 bp of an annotated TSS) were robustly called with confident read depths (Fig 1D).

In the differential analysis of H3K4me3 peaks, 720 proximal peaks were differentially enriched, and these were primarily down-regulated among HD cases relative to controls (104 up-regulated and 616 down-regulated in HD). In contrast, 260 distal peaks were differentially enriched, and these were primarily up-regulated in HD relative to controls (209 up-regulated and 51 down-regulated in HD). These findings suggest that proximal and distal H3K4me3 peaks perform different functions and respond differently to the pathology initiated by the mutant HTT protein.

The H3K4me3 mark is known to associate with active gene transcription, and this relationship is confirmed in this study, where high versus low H3K4me3 proximal peak density and was strongly related to high versus low mRNA expression (Fig 2B, χ^2^ p-value < 2.2 × 10^−16^). It should be noted however, that for the 720+961 “differential” genes, the rho for both HD and controls is reduced, although more in HD (Spearman rho = 0.33) than in controls (rho = 0.4). In contrast however, differentially enriched H3K4me3 proximal peaks were not associated with increased expression of the implicated genes. Few genes with differential enrichment of H3K4me3 density showed differential mRNA levels. The disruption of the regulatory influence of the H3K4me3 enrichment on gene expression in HD brains provides strong evidence for transcriptional dysregulation as a major component in disease expression in HD (see Fig 2B and 2C), as has been previously proposed [9,10], or it may suggest that additional epigenetic marks or mechanisms are required to better explain the full range of transcriptional dysregulation associated with the disease [53].

The genes with the most differentially enriched H3K4me3 marks at their TSS are *ALG14* (Asparagine (N) glycosylation) which regulates protein folding and stability; *LRRTM2 (*leucine rich repeat transmembrane neuronal 2), involved in development of excitatory synapses; *HES4* (Hairy And Enhancer Of Split 4) which is involved in protein dimerization and transcription factor binding; and *COX7B* (Cytochrome C Oxidase Subunit 7B) which is a terminal component of the mitochondrial respiratory chain. Of these only HES4 is differentially expressed, and we have previously described *HES4* as having differential H3K4me3 enrichment and being related to the extent of pathological involvement in the striatum [32].

Other genes shown to have a reduced H3K4me3 signal in HD or corresponding mouse model from a previous study [31] are also revealed in this study. For instance, the down-regulated *Bdnf* gene has reduced H3K4me3 signal at one of its promoters (exon II) in HD and R6/2 mouse. We also observed the same trend (fold change = 0.78, q-value = 0.027). Other genes shown have significant decreases of H3K4me3 in Vashishtha et al. also had decreased H3K4me3 levels in our results, such as PENK1 (fold change = 0.34, but not significant), and DRD2 (fold change = 0.43, q-value = 0.029).

### Differential H3K4me3 enrichment is not associated with differential gene expression

While a genome wide analysis of mRNA levels from our sequencing studies showed a strong correspondence with H3K4me3 enrichment (χ^2^ p-value < 2.2 × 10^−16^; see Fig 2B), this relationship was notably absent for differentially enriched H3K4me3 peaks and differentially expressed genes (DEGs) (see Fig 2C). Notably, 718 (74.7%) of the 961 DEGs are up regulated in HD, suggesting that one might expect to see an increase in differential H3K4me3 enrichment at the TSS of these DEGs. However, only 58 of the 720 differential H3K4me3 proximal peaks are associated with DEGs, and only 20 of these (2.8% of the 720) are in the expected regulatory direction (18 down-regulated DEGs have reduced differential H3K4me3, and 2 up-regulated DEGs have increased differential H3K4me3 enrichment), suggesting that differential H3K4me3 is not responsible for the DEGs. This result holds with a previous study [31] where genome-wide analysis of H3K4me3 and RNAseq in the 12-week cortex of R6/2 mouse revealed a high degree of overlap between genes with decreased expression and decreased H3K4me3 levels (e.g. 293 in down:down, 24 in up:up). Although the mouse data may not be directly comparable, by simply checking the orthologs of the 293 down:down genes in our human data we found that most of these genes also have a down:down relationship (i.e. decreased H3K4me3 levels and decreased expression in HD). While both studies found consistent results for the selected genes and there is a large overlap between genes with same direction change in expression and H3K4me3 levels, nonetheless, a proportion of genes were found not infrequently and unexpectedly in opposite directions in both studies. This discordant pattern holds up in an examination of the concordance of the GO enriched terms for down-regulated DEGs overlap with increased differential H3K4me3 signals described below.

### Analysis of GO term enrichment for genes at enriched H3K4me3 peaks

In an analysis of GO term enrichment only the genes associated with down-regulated peaks in HD showed significant GO terms, with a total of 166 enriched GO terms. We compared the GO terms for differential H3K4me3 with those for the 961 differentially expressed mRNAs (q<0.01). In GO and pathway analysis, we observed similar GO term enrichment in the up-regulated mRNA genes in HD and the down-regulated H3K4me3 target genes in HD.

Although the biological processes of these terms were diverse, organ morphogenesis and positive regulation of gene expression were the most frequently implicated terms. Common molecular function and cellular component GO terms relating to transcription (e.g. Sequence specific DNA binding and RNA pol II activity) and the extracellular matrix (membrane and cell periphery) were observed (see Fig 3). The unexpected pattern of opposite trend of regulation (e.g. increased mRNA levels with decreased H3K4me3) presenting overlapping GO term enrichment, suggests the increased levels of mRNA are likely attributed to regulatory mechanisms other than H3K4me3 control of transcriptional initiation.

Transcriptional dysregulation has a crucial role in HD pathology. Interactions between mutant HTT and important TFs including SP1 [54–56], REST [54,57,58], TAFII130 [56] and others have been observed and may contribute to the observed transcriptional dysregulation [9,59]. Transcriptional dysregulation may also be influenced by the accumulation of the mutant HTT protein. Two recent studies with in vivo and HD Drosophila models showed that the mutant HTT protein facilitates the chromatin regulator PRC2 and alters H3K4me3 states at transcriptionally active genomic loci [60,61].

Several mechanisms might be responsible for the disparity between H3K4me3 enrichment and mRNA levels. Our findings of decreased histone modifications may represent an attempt by the neuron to rescue or reverse via epigenetic means, the aberrant increase in transcription that we see in mRNA levels by RNA-sequencing. Alternatively, decreased H3K4me3 signal has been associated with apoptosis [62] which has been strongly implicated in HD and thus the differences that we observe may represent part of that neurodegenerative process in HD.

### Role of distal H3K4me3 peaks

We subdivided the peaks into proximal (defined as within 1000bp of a transcription start site), and distal peaks (located greater than 1000bp of a transcription start site. One third of H3K4me3 peaks (34.2%) were located distally from transcription start sites, and 260 of them were differentially enriched between HD and control (S4 Table) with 209 up-regulated and 51 down-regulated in HD.

H3K4me3 is usually considered as a promoter mark for actively transcribed genes [26,63–65]. However, several studies support that H3K4me3 can mark enhancers as well [66,67]. In this study we projected the commonly known regulatory marks on H3K4me3 peaks and found that the distal peaks show largely similar patterns of aggregation as proximal peaks, as seen in Fig 5B. One possibility is that some distal peaks are promoters of unannotated genes or non-coding RNAs [68,69]. But this explanation does not appear to account for most distal peaks for the following reasons: First, if the distal H3K4me3 signal is an active promoter for an unannotated gene, one may expect a corresponding increase RNA-seq reads derived from the transcription at that position. However, most distal peaks have very low RNA-seq read coverage (0 or 1 read, see S5 Fig). Second, in our CAGE tag directionality analysis, distal peaks show a clear pattern of bidirectionality, unlike the uni-directionality for proximal peaks. This finding is consistent with the defining feature of transcribed enhancers (or eRNAs) recently characterized by the FANTOM consortium [42]. By intersecting distal peaks with three known sets of enhancers, an estimated 36% of the distal H3K4me3 peaks appear to localize to enhancer sites.

It is worth noting that the distal peaks are generally less intensive than proximal peaks (Fig 1D). Although we had previously checked the specificity of the anti-H3K4me3 antibody we used, for absence of detectable immunoreactivity for the related epitopes of H3K4me2 and H3K4me1 [70], future studies are needed to explore whether the antibody detects other types of histone modification at distal peaks.

### TF differentially bound in distal peaks

We compared the TFs bound at the proximal and distal peaks, and found that the TFs associated with chromatin structuring are enriched in distal peaks. Chromatin looping allows far-distant enhancers to be brought into the proximity of promoters of protein-coding genes. Enhancers are thought to interact with protein complexes necessary for chromosome looping and to facilitate RNA Polymerase II recruitment of the promoter of the target gene. This result is consistent with the finding from a recent study using ChIA-PET interaction data that cohesin, CTCF, and ZNF143 are the key components of 3D chromatin structure [71]. Studies in peripheral tissues found that many of the ectopic H3K4me3 sites corresponded to enhancers bound by CTCF and cohesin [66,67]. Recently the link between distal H3K4me3 peaks and promoter-enhancer loopings has also been reported for human cerebral cortex [72,73].

When looking at the enriched TFs in differentially enriched H3K4me3 peaks between HD and control, we found six transcription factors have enriched binding sites in HD-specific distal peaks, including EZH2 and SUZ12. Previous studies also found the role of EZH2 for DNA hypermethylation in the epigenetic inhibition of the dopamine receptor D4 (DRD4), which is important for neurodegenerative disease such as HD and Parkinson’s disease [74]. These findings may implicate the enrichment of EZH2 in the HD-specific distal H3K4me3 peaks.

EZH2 and SUZ12 are also core subunits of the polycomb repressive complex 2 (PRC2). Given that PRC2 should recognize the mark that EZH2 generates, the finding that PRC2 is depleted while EZH2 is enriched suggests that the H3K4me3 mark may appear preferentially on those EZH2 target regions that are not regular targets of the PRC2 complex.

H3K4me3 was recently found to inhibit PRC2 assisted by the Su(z)12 C terminus [50]. Our findings of depleted PRC2 in HD-enriched H3k4me3 peak regions may indicate that increased H3K4me3 in HD is associated with PRC2 inhibition.

In summary, we examined the differential enrichment of H3K4me3 signals and the relationship of this histone mark to RNA levels in both HD and control prefrontal cortex. Genes with differential H3K4me3 signal at their promoters were rarely found to be differentially expressed. These findings support the hypothesis that transcriptional dysregulation plays an important role in the pathogenesis of HD. GO term analysis of the genes with differential H3K4me3 enrichment identified terms only among the set of genes with down-regulated H3K4me3 in HD, and most frequently implicated biological process terms with organ morphogenesis and positive regulation of gene expression. More than 9000 H3K4me3 peaks were located not near to any recognized transcription start sites and approximately 36% of these “distal” peaks co-localized to known enhancer sites. Transcription factors enriched in distal peaks, implicated the polycomb repressive complex 2 (PRC2), suggesting that H3K4me3 histone hypermethylation is linked to dysregulated polycomb activity in the HD neuronal epigenome.

## Materials and Methods

### H3K4me3 ChIP-seq sample preparation

Postmortem brain tissues from the prefrontal cortex Brodmann area 9 (BA9) were obtained from the Harvard Brain and Tissue Resource Center (HBTRC), McLean Hospital, Belmont MA. Six human HD post-mortem samples were selected for ChIP-seq. HD cases were confirmed to have expanded CAG repeats longer than 40 (mean = 44.3), a mean age at death of 60.3 years, and a mean postmortem interval of 20.1 hours. Six non-neurological controls of similar age (mean = 67.7), sex, and postmortem interval (mean = 19.6) were selected from samples studied previously in the Akbarian Lab [33–35] for H3K4me3 ChIP-sequencing (Table 1). This study has been designated exempt (Protocol # H-28974) by the Boston University School of Medicine Institutional Review Board, as no human subjects were studied and all data are derived from post-mortem human brain specimens.

Neuronal nuclei from prefrontal cortex BA9 were NeuN+ labeled and fluorescence activated sorted (FACS) for subsequent H3K4me3 ChIP-seq. Briefly, approximately 300 mg to 500 mg of -80°C frozen grey matter dissected from BA9 was homogenized in 5 ml lysis buffer and ultracentrifuged over sucrose solution at 24,400 rpm for two hours at 4°C. The resulting pellet was suspended in 550 μl PBS, and immunolabeled with NeuN for 60 minutes while rotated in darkness. NeuN+ and NeuN- fractions were separated and collected by FACS [52].

ChIP sequencing was performed for the NeuN+ fraction. Briefly, genomic regions with H3K4me3 modification were extracted by chromatin immunoprecipitation by incubating digested nuclei with anti-H3K4me3 (Upstate; cat # 07–473) at 4°C overnight and further prepared into ChIP-seq library for sequencing, as described previously [75]. For each brain sample, 1 μg of DNA was used to construct sequencing libraries using Illumina TruSeq Sample Prep Kit, according to the manufacturer’s protocol (Illumina, San Diego, CA), and sequenced using 51nt single-end reads on Illumina’s GA II. The ChIP-seq data for six Huntington’s disease patients and six controls from this study have been submitted to GEO (http://www.ncbi.nlm.nih.gov/geo/) under the accession number of GSE68952.

### H3K4me3 ChIP-seq read mapping and peak detection

We mapped all reads in H3K4me3 ChIP-seq libraries to the human reference genome (hg19) with Bowtie (version 1.0.0) [76] allowing 1 mismatch (see Table 1 for mapping statistics). Mapped reads were used for the input of the MACS algorithm (version 1.4.0rc2) [77] to identify regions in the genome that are enriched in H3K4me3 (called H3K4me3 peaks or peaks in short). We extracted H3K4me3 peaks with false discovery rate (FDR) ≤ 0.01. Peaks that were called in fewer than half the HD samples or fewer than half the controls were removed from that group respectively. We then merged the remaining peaks from each group to create a union set of peaks for all subsequent analyses. Thus some peaks were called in only the HD group, while others were called only among controls.

The union set of peaks were designated as “proximal” if the distance between the central points of peaks to the nearest transcription start site (TSS) of a gene is less than or equal to 1 kb, otherwise they were designated as “distal” peaks. We further annotated the peaks as either “common” or “unique” based upon the fold change of H3K4me3 signal at the peak summit between HD and control: common peaks have less than or equal to 2 fold change, and unique peaks have greater than 2 fold change. We defined the H3K4me3 signal at a peak by first taking the average of the RPM (reads per million) at the summit position over samples in HD and control, then normalizing the peak signals across the HD and control groups based on the “invariant set” method [78] (see S1 Table for summary of detected peaks).

### RNA-seq data

Analyses reporting differential expression of mRNA in HD versus controls are reported elsewhere (Labadorf et al. submitted). DESeq2 [79] was used to identify differentially expressed genes (DEGs) between HD and control, adjusting for age at death binned into intervals 0–45, 46–60, 61–75, and 90+ and a categorical RNA Integrity Number (RIN>7 and RIN≤7) as covariates. Differential expression analysis identified 961 out of 27,907 confidently expressed genes with significantly altered expression at *q*-values ≤ 0.01 and abs[log_2_(fold change)] ≥ 1 in 20 HD vs. 49 control samples from the BA9 region of the prefrontal cortex. The list of all 961 DEGs is in S2 Table. Sample information is provided in Table 1 and the methods to define DEGs are found in (Labadorf et al. submitted).

To correlate gene expression with H3K4me3 signal at promoters, we extracted the normalized counts from DESeq2 (e.g. counts(dds, normalized = TRUE)) and further normalized the values by the total length of meta-exons of annotated genes. Meta-exon were defined by merging all overlapping exons of a gene.

### Detection of differentially enriched H3K4me3 peaks

We extracted ChIP-seq reads mapped to the promoter regions (defined as ±1000 bp of TSS) for all annotated transcripts (Gencode v17) [80] using the coverageBed tool in bedtools (version 2.19.0) [81]. We then applied the DESeq2 algorithm (version 1.4.5) [79] to detect transcripts with differential H3K4me3 signals at promoters between HD and control (q-value ≤ 0.01 and abs[log_2_(fold change)] ≥ 1). For genes with multiple expressed transcripts, we selected the transcript with the lowest q-value from the DESeq2 result for subsequent analyses, in order to match with the differentially expressed genes from RNA-seq. A list of 720 genes with differential H3K4me3 proximal peaks is in S3 Table.

For distal peaks, we extracted the total reads mapped to each peak across all samples and used DESeq2 to compute enrichment of H3K4me3 signal between HD and control. Using the same cutoff as for promoters, we defined 260 distal peaks with significant enrichment and this list is in S4 Table.

### Gene ontology analysis

Using GOseq (version 1.16.2) [82], we identified significantly enriched gene ontology (GO) terms for the following sets of genes: (1) the genes that correspond to the proximal differential H3K4me3 peaks and (2) the nearest genes to the unique proximal peaks. To compare these results with differentially expressed genes between HD and control, we also computed the GO term enrichment (q-value ≤ 0.01) for the 961 DEGs (Labadorf et al. submitted). We used REVIGO web server [83] to summarize and reduce the complexity of the significant GO terms (*simRel* score cutoff = 0.7).

### Pathway analysis

We identified significantly enriched pathways with SPIA (version 2.16.0) [41] for the genes that corresponded to the proximal differential H3K4me3 peaks. We then extracted the sub-pathways containing the genes with KEGGgraph [84]. We also applied the same analysis to the set of 961 DEGs (Labadorf et al. submitted).

### Transcription factor enrichment in H3K4me3 peaks

Transcription factor (TF) binding regions were extracted from the TFBS cluster file (wgEncodeRegTfbsClusteredV3) downloaded from the UCSC genome browser [85], which correspond to peaks called from ChIP-seq data for 161 TFs in 91 cell types performed by the ENCODE consortium [86,87]. We then intersected the H3K4me3 peaks with the TF binding regions and determined the number of TFs found in each category of H3K4me3 peak. Each overlapping TF was counted only once, regardless of the number of different cell types in which it was found. We then compared the number of occurrences of TFs between the two categories of H3K4me3 peak by Fisher’s exact test, and corrected the resulting p-values for multiple testing using the Benjamini-Hochberg procedure. Odds ratios (OR) were estimated from Fisher’s exact test. TFs with q-value < 0.01 and odds difference >30% (i.e. OR >1.3 or OR<0.7) were deemed significant.

### Enrichment of other regulatory signals with H3K4me3 peaks

In addition to TF binding data from the ENCODE consortium, we also compared our H3K4me3 peaks with the regulatory signals from the following three sources: (1) CAGE signal in the frontal lobe adult brain from the FANTOM5 project [42], (2) H3K4me1, H3K4me3, H3K27ac, and H3K9ac ChIP-seq signal in the middle frontal lobe of brain, and (3) DNase I signal in human fetal brain downloaded from the Roadmap Epigenomics project [49]. For each H3K4me3 peak, we first extended to 1000bp in length centered at the maximum value of overlapped DNase narrowPeak (if any) or the midpoint of the H3K4me3 peak. Then we subdivided each peak into 200 bins (i.e. 5bp per bin) and computed the mean values of the signal in each bin, which were then used to create the aggregation plots in Fig 5B. The transcription directionality (in Fig 5C) of each peak was computed using the same definition as that used in the FANTOM5 project [42]. Briefly, the normalized CAGE signals from first half bins (i.e. 1–100 bins) and second half bins (101–200 bins) were used to calculate a peak-set wide directionality score, *D*, for each peak over aggregated normalized reverse, *R*, and forward, *F*, strand CAGE signals across all peaks, D = (F-R) / (F+R).

To investigate whether our distal H3K4me3 peaks may function as enhancers, we compared them with three sets of enhancer annotations: (1) the ‘strong’, ‘weak’, or ‘bivalent’ enhancers based on genome-wide chromatin states of the brain dorsolateral prefrontal cortex tissue generated by the Roadmap Epigenomics consortium [49], (2) the super enhancers in brain mid-frontal lobe defined by occupancy of the master transcription factors and mediators [88], and (3) the transcribed enhancers defined based on the bidirectional CAGE signal from the FANTOM5 project [42].

## Supporting Information

S1 Fig Flowchart of H3K4me3 peak calling. This figure depicts the process by which the ChIP-seq data were aligned and processed to identify proximal and distal peaks, as well as differentially expressed common and unique peaks.
(TIF)

S2 Fig (A) The number of HD and control samples supporting a called H3K4me3 peak. Most peaks (59.2%) were called in all six HD and control brains. The area of the bars corresponds to the number of peaks. Only peaks with at least 3 samples supported in either of the groups are included in this plot and analyzed in this study. (B) The number of samples supporting a H3K4me3 peak and its normalized signal in each group. Peaks called in all six HD and all six controls had higher peak densities than did peaks called in fewer than six.
(TIF)

S3 Fig (A) mRNA levels are bimodal for both HD and control, and consequently we subdivided then into “high” and “low” groups (dotted line). (B) The distributions of both HD and control H3K4me3 peaks are bimodal and consequently we subdivided them into “high” and “low” groups. (C) Histogram of H3K4me3 mean normalized signals in HD cases (left) and controls (right). Bars are colored according to the high (red) or low (blue) expression level. Both HD and control cases show a similar level of correspondence between expression and H3K4me3 level.
(TIF)

S4 Fig GO terms enriched with genes up-regulated in HD. The GO terms overlapped with those of genes with down-regulated H3K4me3 peaks are highlighted in red.
(TIF)

S5 Fig The distribution of expression level of distal peaks, measured as the median of RNA-seq reads summit in the distal peaks across the samples in HD and controls, shows that most distal peaks are covered with no (0) or low (e.g. <10) RNA-seq reads. (TIF)

S1 Table Detected H3K4me3 peaks in control and HD. CT: Control. Distance to TSS: Distance is measured from the center of each peak to a TSS of the nearest gene. If the peak center is located upstream (5′) of TSS, the distance is minus; otherwise, plus. Location: If a peak overlaps with any gene in GENCODE v17, it is categorized as intragenic; otherwise, intergenic. Note that a peak may be located intragenic of a gene, but it may refer to a different gene with the nearest distance to TSS.
(XLSX)

S2 Table List of 961 differentially expressed genes. SE: Standard error.
(XLSX)

S3 Table List of 720 genes with differential H3K4me3 signal in the promoter regions. SE: Standard error.
(XLSX)

S4 Table List of 9772 distal peaks. 260 distal peaks with differential H3K4me3 signal are marked as “Yes” in the DE-H3K4me3 column. 3560 distal peaks overlapping at least one enhancer sets are marked as “Yes” in the “putative enhancer peak” column. SE: Standard error.
(XLSX)

S5 Table Pathways enriched with genes with differential H3K4me3 signals. In the link to the detected KEGG pathways, genes with both up-regulated and down-regulated differential H3K4me3 signals in HD are highlighted in red. #Gene: Statistics of detected genes (left) and total genes (right) in a pathway. Nested detected pathways: Pathways detected in this analysis and embedded in other detected pathways.
(XLSX)

S6 Table Pathways enriched with differentially expressed genes. In the link to the detected KEGG pathways, up-regulated and down-regulated genes in HD are highlighted in red. #Gene: Statistics of detected genes (left) and total genes (right) in a pathway. Nested detected pathways: Pathways detected in this analysis and embedded in other detected pathways.
(XLSX)

S7 Table List of genes enriched in detected pathways. (XLSX)
