# Supplementary figures and images for: The Role of H3K4me3 in Transcriptional Regulation Is Altered in Huntington’s Disease
Source: PLoS One. 2015 Dec 4;10(12):e0144398. doi: 10.1371/journal.pone.0144398 (PMC4670094; doi:10.1371/journal.pone.0144398)

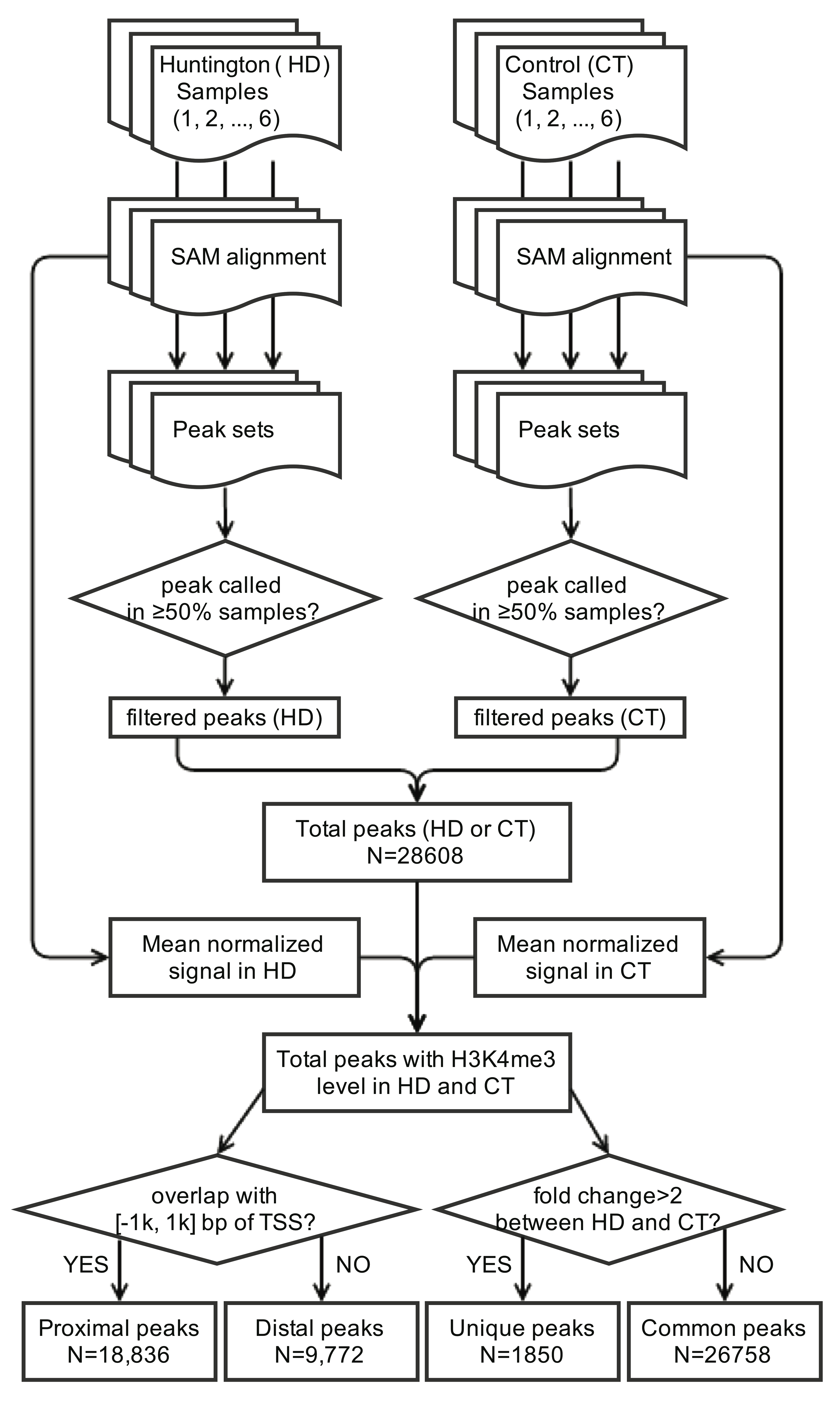

Supplement: S1 Fig — This figure depicts the process by which the ChIP-seq data were aligned and processed to identify proximal and distal peaks, as well as differentially expressed common and unique peaks. (TIF) [file pone.0144398.s001.tif]

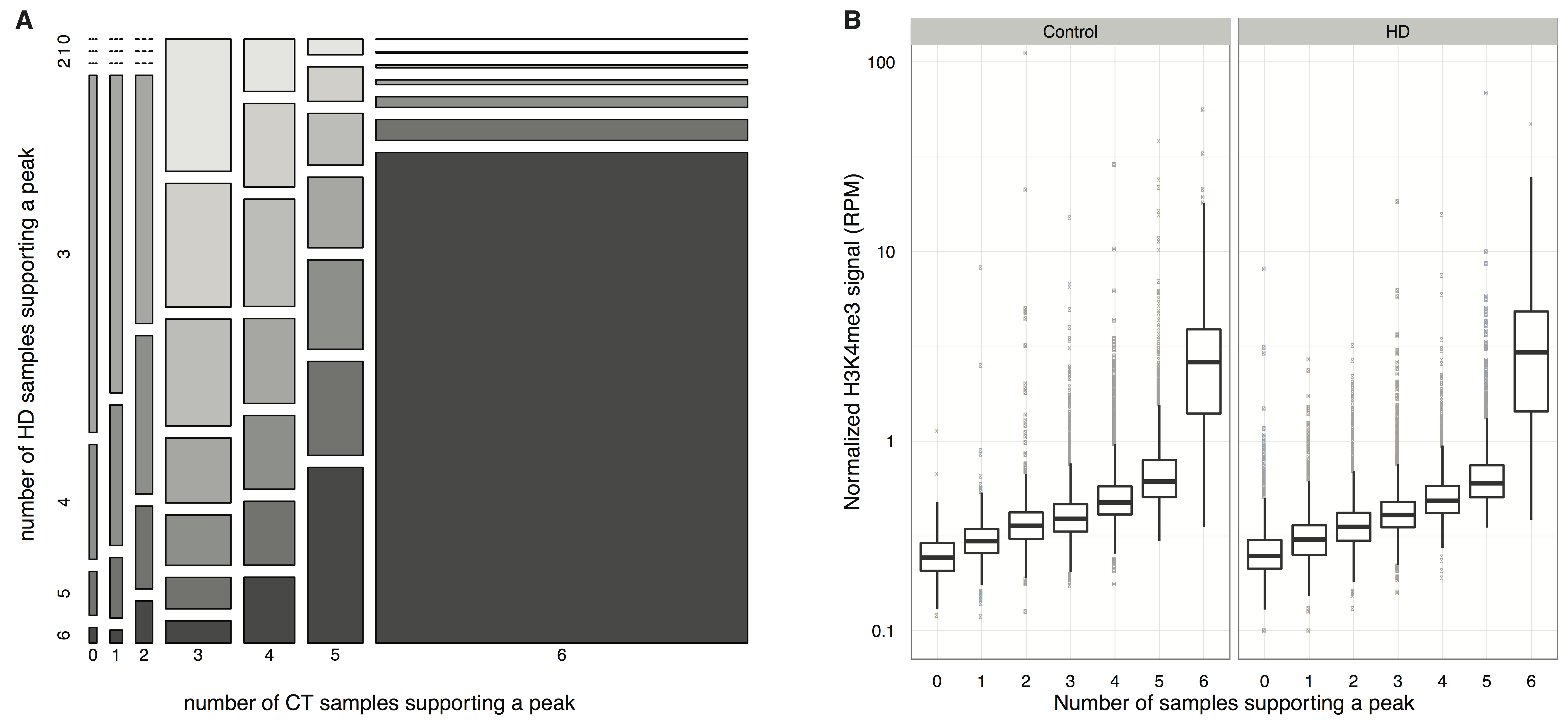

Supplement: S2 Fig — Peaks called in all six HD and all six controls had higher peak densities than did peaks called in fewer than six. (TIF) [file pone.0144398.s002.tif]

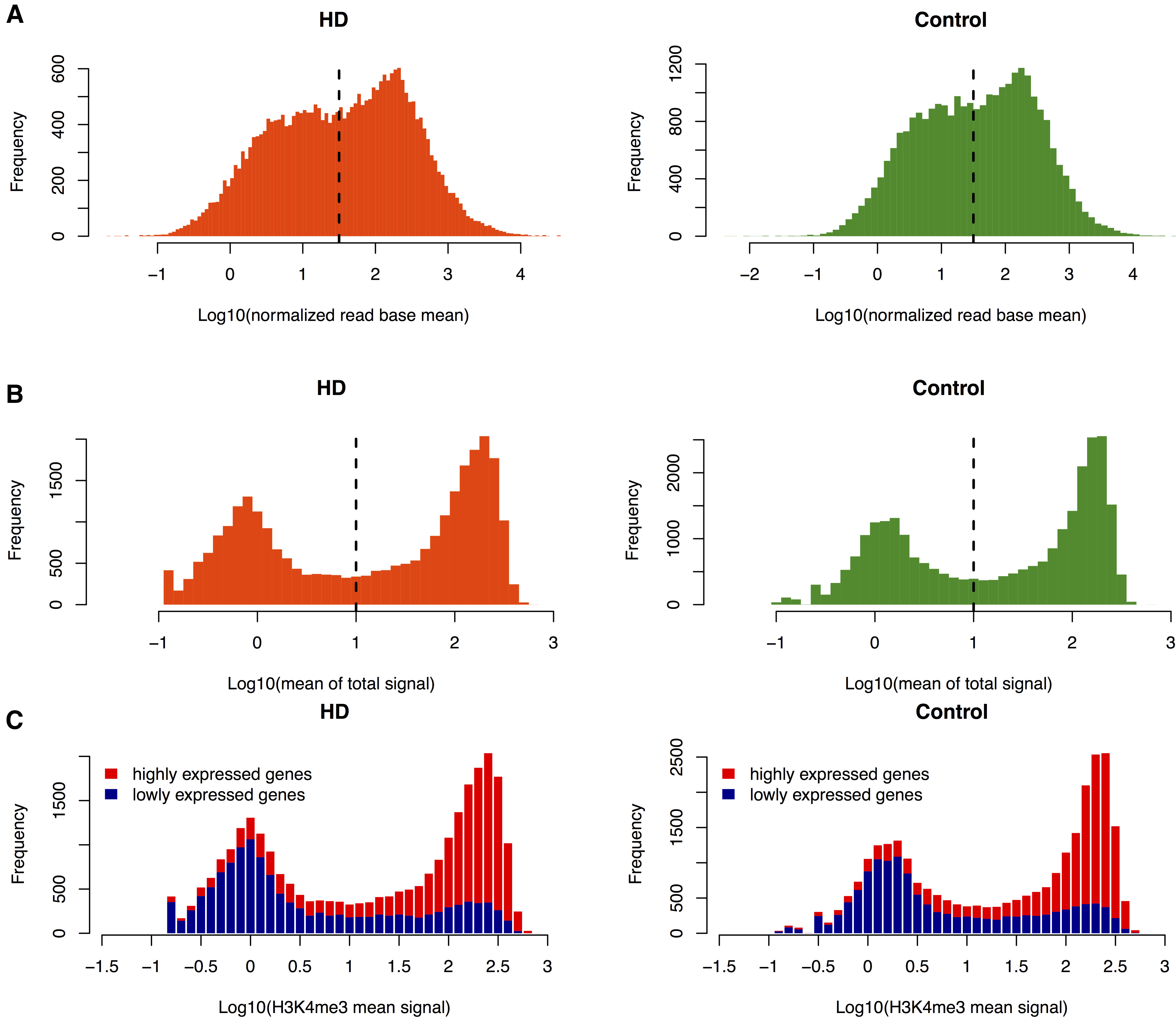

Supplement: S3 Fig — Bars are colored according to the high (red) or low (blue) expression level. Both HD and control cases show a similar level of correspondence between expression and H3K4me3 level. (TIF) [file pone.0144398.s003.tif]

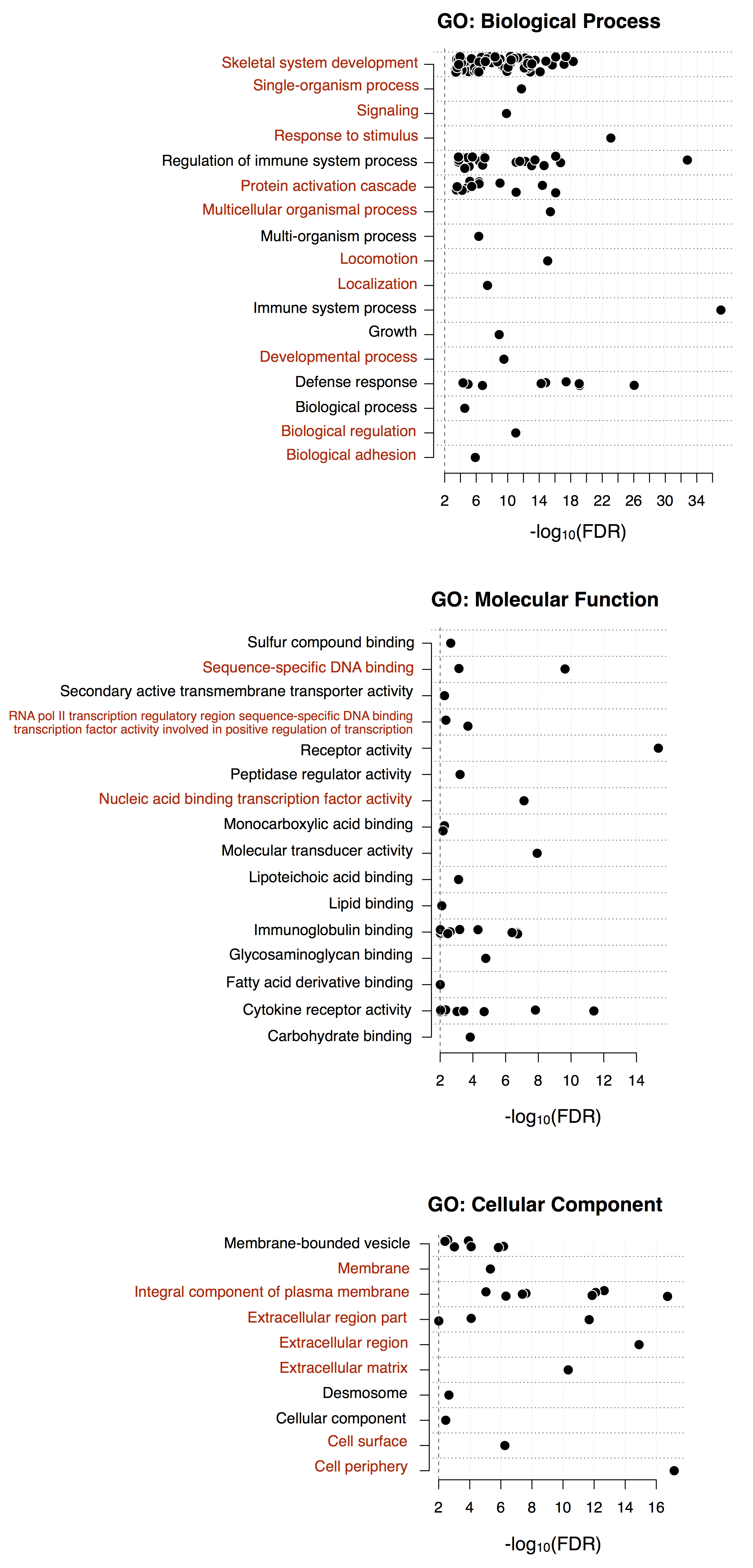

Supplement: S4 Fig — The GO terms overlapped with those of genes with down-regulated H3K4me3 peaks are highlighted in red. (TIF) [file pone.0144398.s004.tif]

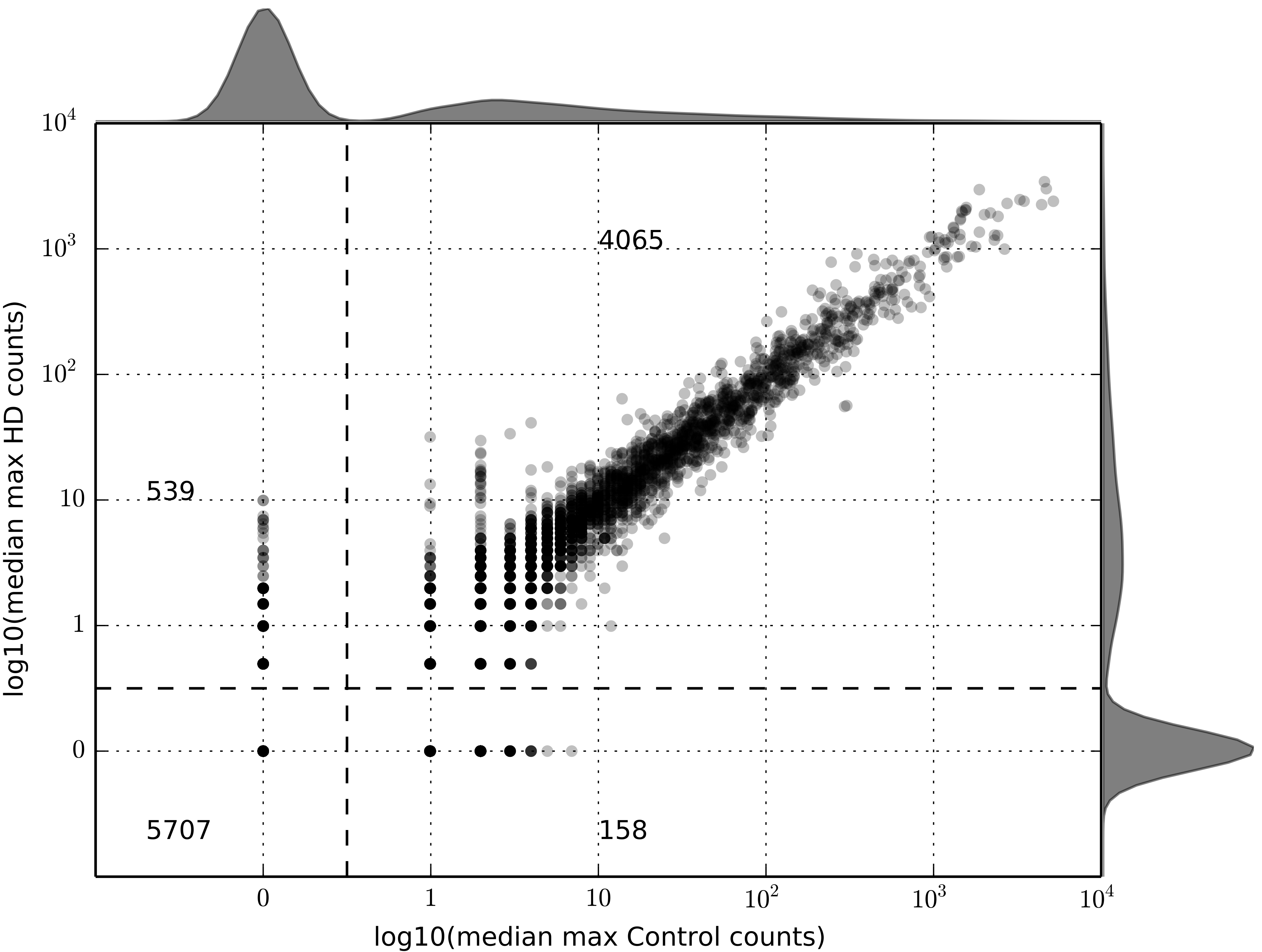

Supplement: S5 Fig — (TIF) [file pone.0144398.s005.tif]
